# Impact of Chronodisruption during Primate Pregnancy on the Maternal and Newborn Temperature Rhythms

**DOI:** 10.1371/journal.pone.0057710

**Published:** 2013-02-28

**Authors:** María Serón-Ferré, María Luisa Forcelledo, Claudia Torres-Farfan, Francisco J. Valenzuela, Auristela Rojas, Marcela Vergara, Pedro P. Rojas-Garcia, Monica P. Recabarren, Guillermo J. Valenzuela

**Affiliations:** 1 Programa de Fisiopatología, Facultad de Medicina, Universidad de Chile, Santiago, Chile; 2 Departamento de Ciencias Fisiológicas, Facultad de Ciencias Biológicas, Pontificia Universidad Católica de Chile, Santiago, Chile; 3 Instituto de Anatomía, Histología y Patología, Facultad de Medicina, Universidad Austral de Chile, Santiago, Chile; 4 Department of Women’s Health, Arrowhead Regional Medical Center, San Bernardino, California, United States of America; Hôpital du Sacré-Coeur de Montréal, Canada

## Abstract

Disruption of the maternal environment during pregnancy is a key contributor to offspring diseases that develop in adult life. To explore the impact of chronodisruption during pregnancy in primates, we exposed pregnant capuchin monkeys to constant light (eliminating the maternal melatonin rhythm) from the last third of gestation to term. Maternal temperature and activity circadian rhythms were assessed as well as the newborn temperature rhythm. Additionally we studied the effect of daily maternal melatonin replacement during pregnancy on these rhythms. Ten pregnant capuchin monkeys were exposed to constant light from 60% of gestation to term. Five received a daily oral dose of melatonin (250 µg kg/body weight) at 1800 h (LL+Mel) and the other five a placebo (LL). Six additional pregnant females were maintained in a 14∶10 light:dark cycles and their newborns were used as controls (LD). Rhythms were recorded 96 h before delivery in the mother and at 4–6 days of age in the newborn. Exposure to constant light had no effect on the maternal body temperature rhythm however it delayed the acrophase of the activity rhythm. Neither rhythm was affected by melatonin replacement. In contrast, maternal exposure to constant light affected the newborn body temperature rhythm. This rhythm was entrained in control newborns whereas LL newborns showed a random distribution of the acrophases over 24-h. In addition, mean temperature was decreased (34.0±0.6 vs 36.1±0.2°C, in LL and control, respectively P<0.05). Maternal melatonin replacement during pregnancy re-synchronized the acrophases and restored mean temperature to the values in control newborns. Our findings demonstrate that prenatal melatonin is a *Zeitgeber* for the newborn temperature rhythm and supports normal body temperature maintenance. Altogether these prenatal melatonin effects highlight the physiological importance of the maternal melatonin rhythm during pregnancy for the newborn primate.

## Introduction

Disruption of the mother’s environment is a key contributor to offspring diseases and conditions that account for approximately one third of the global burden of disease in both developed and developing countries [1]. In this context, disturbance of the temporal organization of physiology and behavior (chronodisruption) accompanying shift work is associated with increased risk of miscarriage, preterm delivery and low birth weight [2]–[4]; both strong predictors of chronic disease later in life [5]–[6].

The orderly day/night alternation is an important signal for appropriate time-of-day organization of physiological functions in mammals [7]. Most physiological functions present 24-h rhythms (circadian) driven by a system of biological clocks comprising a master clock located in the suprachiasmatic nucleus of the hypothalamus, (SCN) and peripheral circadian clocks located in most tissues of the body. The SCN, connected to the retina by the retinohypothalamic tract, entrains to the light/dark cycle and relays this information to internal peripheral clocks through a number of circadian signals, some of them neurohumoral [8]. Several authors have proposed that the circadian rhythm of body temperature provides a global internal entrainment signal for adult peripheral clocks [9]–[10]. There is limited information on the effects of chronodisruption in the circadian system in pregnant primates or on its effect on the offspring.

The presence of a synchronized circadian temperature rhythm soon after birth in human and non-human primate newborns allows for testing the *in vivo* effects of maternal chronodisruption during pregnancy in the offspring [11]–[13]. The early onset of the temperature rhythm suggests that circadian thermoregulation was entrained prenatally. Prenatal entrainment by maternal signals of newborn circadian behavioral rhythms (activity, drinking) appearing weeks after birth has been demonstrated in rodents [14]–[15]. Entrained circadian clock genes oscillate in fetal organs in the capuchin monkey, rat and mouse thus conceptually, fetal organs could be considered as maternal peripheral circadian clocks [16]. As fetuses, newborns were obligatorily exposed to the maternal circadian rhythm of temperature and to circadian signals derived from the maternal activity/rest rhythm, both tuned to the LD cycle. In addition, as fetuses, newborns were exposed to the rhythm of maternal melatonin, hormone that crosses the placenta unaltered [17]–[19]. This rhythm is suppressed by chronic exposure to constant light in pregnant rhesus and capuchin monkeys [20]–[21]. We hypothesize that any of these maternal circadian rhythms could potentially convey prenatal light/dark (LD) cycle information for the temperature rhythm in the primate newborn. Therefore, we investigated the effect of exposing pregnant capuchin monkeys to maternal chronodisruption (constant light from the last third of gestation to term), on maternal temperature and activity rhythms and the temperature rhythm of the capuchin newborn. To assess the possible role of melatonin as a *Zeitgeber* on these rhythms, a daily dose of melatonin was given at 1800 h to a group of pregnant females exposed to constant light.

## Experiments and Methods

### Animals

Pregnant capuchin monkeys and newborns from the Primate Center, Pontificia Universidad Católica de Chile, were used in the experimental protocols described below. The pregnant females were maintained in individual cages in a room with constant environmental temperature and humidity (27°C and 70%, respectively) and a 14∶10 light:dark schedule during the first 100 days of gestation (term 158 days). Water was supplied *ad libitum*. Feeding consisted of fresh fruits, eggs, pellets and biscuits (798.2 kcal *per* day and 27.6 g of proteins *per* day); and meals were given at 1200 and 1800 h. At 100–101 days of gestation, the animals were removed to another room and assigned to the chronodisruption protocols. General conditions and feeding times were maintained as before. Care during pregnancy was performed as described [21]. All routine maternal procedures were performed under i.m. ketamine (10 mg/kg of body weight; Ketaset, Laboratorios Wyeth Inc., Santiago, Chile). Pregnancy length was recorded. Upon delivery, mothers and newborns were examined by the colony veterinarian, weighed and measured. All pregnancies ended in spontaneous term delivery. The newborns remained with their mothers and were fed by them except during newborn temperature measurements. Upon completion of the experimental protocols, all newborns were returned to their mothers and the mother infant pair returned to the colony. Experimental Protocols were approved by the Bioethics and Biosafety Committee of the Faculty of Biological Sciences, Pontificia Universidad Católica de Chile (FONDECYT-LINEAS COMPLEMENTARIAS 1998-8980006).

### Experimental Protocol

To induce maternal chronodisruption, pregnant females were exposed to constant light from about 100 days of gestation up to the post-delivery conclusion of the experiment, as previously described [21]. Ten pregnant females were maintained in a constantly lit environment (2000 lux at the head level). Five of them received a teaspoon of fruit juice daily at 1800 h as placebo (LL group) for 51.8±4.7 days and the other five (LL+Mel group) received 250 µg/kg body weight of melatonin (Maver Ltda. Laboratory, Santiago, Chile) in fruit juice daily at 1800 h for 46.0±1.8 days. Six additional females remained in 14∶10 h LD (lights on at 0700 hours, LD, control group). All pregnancies resulted in clinically healthy full term newborns that were reared by their mothers in the LL or LD photoperiod used during gestation. On a previous study [21] we demonstrated that constant light exposure during pregnancy effectively suppressed the maternal plasma melatonin rhythm in the capuchin monkey [21]. LL females maintained lower melatonin concentration throughout pregnancy (measured every ten days at 1100 h) than LD females. We also found that daily oral melatonin treatment resulted in plasma melatonin values at 1100 h twice as high those in LD females [21]. We have preliminary information on the levels of melatonin attained soon after melatonin ingestion from two LL+Mel animals of another study. In these, values at 4 hours after melatonin intake were 10 fold higher than the nocturnal melatonin peak reported in LD females (unpublished data). Confirming findings reported in [21], in the present study, LL or LL+Mel treatment had no effect on pregnancy outcome or newborn weight (Table 1).

**Table 1:** Duration of the maternal treatments during gestation and newborn outcome.

Duration of maternaltreatments (days)				Newborn		
	N	LL	Mel	GA at birth(days)	Weight(g)	SexM/F
LD	6	−	−	157.4±2.0	217.5±9.2	3/3
LL	5	51.8±4.7	−	154.2±2.2	213.0±14.5	3/2
LL+Mel	5	51.4±2.2	46.0±1.8	156.2±1.2	232.0±12.1	2/3

Data are mean±SE. Maternal treatments: LD: photoperiod 14∶10 during pregnancy and post delivery, lights on at 0700 hours; LL: continuous light during pregnancy and post delivery, LL+ M: continuous light during pregnancy+daily dose of melatonin at 1800 hours until delivery followed by continuous light post delivery. M = male; F = female GA = Gestational age.

Activity and temperature measurements using subcutaneous telemetry transmitters were performed in females during the last week of pregnancy and early postpartum. Temperature of their newborns at 4–6 days of age was also assessed by telemetry. In pregnant females, a sterile calibrated transmitter (VM-FH-BB, disc 30 mm diameter and 15 mm thickness, Mini-Mitter Co.) was implanted in the flank under i.m. ketamine anaesthesia (10 mg/kg body weight) and sterile conditions 10–6 days before the anticipated term. Post-operative care included wound inspection and cleaning and initial analgesia [22]. In the newborn, a transmitter (VM-FH disc, 20 mm diameter and 10 mm thickness, Mini-Mitter Co.) was implanted in the flank at 2–3 days of age using the same procedures. An independent group of 10 4–6 days old newborns from non-instrumented mothers kept and raised in LD conditions conditions was used to validate telemetric measurement of temperature against rectal temperature. There were no differences in mesor, amplitude or acrophase between body temperature and rectal temperature rhythms (Table S1).

Maternal telemetric recordings were performed by attaching the receiver to one of the inside walls of the cage. Data were collected at 15-min intervals over 8–10 days. After 48–72 hours postpartum the maternal transmitter was surgically removed and postoperative care was performed as described above.

Telemetric temperature recordings were performed in newborns separated from the mother. The day of recording, newborns were taken to another room and placed in a plastic cage warmed with a heating blanket and containing a furry teddy bear, to which the newborns clutched. The telemetry receiver was placed under the cage. Bottle-feeding was provided every two hours (NAN 1, Nestle, Vevey, Switzerland). Telemetric data were collected at 15-min intervals over 24-h, starting at 0800 h. The room maintained the respective photoperiod to which the mothers were exposed during pregnancy and postpartum. In all LD experiments, a dim red light (<0.2 lux) was left on during the night. After completion of the measurements, the transmitter was removed and the newborns were reared by their mothers in the normal photoperiod of the colony.

### Data Analysis

Data are expressed as means ± S.E.M. Radiotelemetric recordings containing 15-min data collections from each individual were analysed by Cosinor using the DATACOL 3 program (Mini-Mitter Co., Sunriver OR). This method tests whether the data fit the cosine function with a period of 24-h, V_t_ = M+A cos 15 (t−Φ); with V_t_ being the value of the variable (temperature, activity) at time t; Φ the acrophase (hour at which the variable reaches the maximum value); M, the mesor (average of the variable over 24-h) and A, the amplitude (difference between the value of the variable at Φ and the mesor). Parameters (mesor, amplitude) of the Cosinor equations fitting 24-h rhythms (*P*<0.05) in individuals were compared by ANOVA and Tukey’s test. Randomness of the acrophases for the temperature rhythm was tested by Rayleigh’s test and comparison of acrophases between groups was performed by Watson-Williams test [23]. In addition data obtained at hourly intervals was analysed by ANOVA for repeated measures and Tukey’s test. Statistical analyses were performed using GraphPad Prism software (version 3.02; GraphPad Software Inc. San Diego, CA). Results were considered significant when P values were <0.05. When data indicated significant differences in the 24-h, it was fitted to a theoretical cosine function using the same software.

## Results

### Effects of Maternal Chronodisruption during the Last Third of Gestation on the Rhythms of Temperature and Activity in the Mother

The main effect of chronic constant light exposure in the pregnant capuchin was a change in the acrophase of the locomotor activity rhythm altering the relationship between locomotor activity and temperature rhythms. We did not observe changes in maternal food intake or weight gain. Pregnancy duration and newborn weight were not affected by the treatments (Table1).

**Figure 1:**
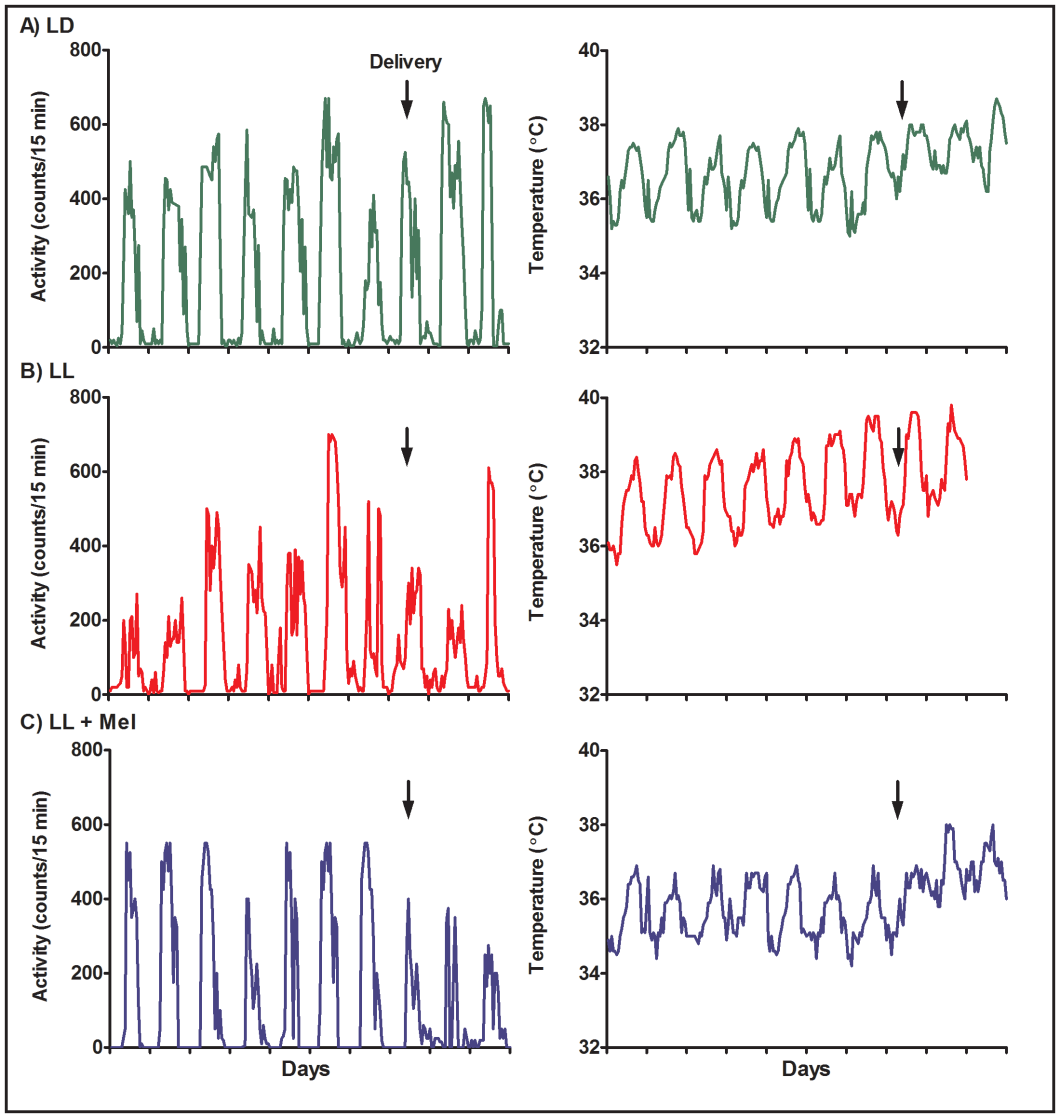
Representative examples of circadian rhythms of activity (left panel) and temperature (right panel) in three pregnant capuchin monkeys. LD: females maintained in light:dark 14∶10 during pregnancy; LL: females maintained in constant light during pregnancy; LL+M: LL females receiving a daily melatonin replacement at 1800 h. The arrows indicates delivery.

Activity and temperature rhythms were detected in all pregnant females, before and after delivery (Figure 1). Regardless of the treatment, the day preceding delivery, and one day postpartum, some females showed decreased activity and tended to spend most of the day in a corner of the cage, usually away from the telemetry receiver, resulting in missing data points in the record. Activity resumed to values observed before delivery in the following postpartum days. Temperature increased during the first 48 h following delivery in most females although comparison of mesor before and after delivery did not reach significance. Activity and temperature rhythms were calculated from the 24-h recordings of each rhythm obtained 96 hours before delivery in order to compare animals in the same condition.

In the three groups of pregnant females activity rhythms and temperature rhythms showed a non random distribution of acrophases (P<0.05, Rayleigh’s test) suggesting entrainment to external zeitgebers. In LD, locomotor activity started at about 0900 h, coinciding with the initiation of housekeeping procedures in the colony. Activity increased when the first meal was presented (1200 h) and was minimal during lights off. The dispersion of acrophases was very small (Table 2). Exposure to constant light delayed the start of activity, and increased the dispersion of acrophases compared with LD, nevertheless under LL activity acrophases still showed a no-random distribution in the 24-h, the mean acrophase being positioned between the 1200 and 1800 h meals (Table 2).

**Table 2:** Parameters of the cosinor equations describing the pregnant capuchin circadian locomotor activity and body temperature rhythms 96 hours before delivery and those of the temperature rhythm in their newborns (mean ± SEM).

Maternal rhythms (96 hours before delivery)									Newborn (4–6 days of age)			
Locomotor activity (counts/h)					Temperature°C				Temperature°C			
	Mesor	A	Φ (hours)	Mesor		A	Φ (hours)	Mesor		A	Φ (hours)	
LD	329.2±70.5	392.6±91.5	12.9±0.2	37.1±0.3		0.9±0.1	16.7±0.5**	36.1±0.2		0.74±0.1	17.0±0.5(15.8–19.1)	
LL	233.0±87.6	321.8±94.3	16.8±1.2*	37.2±0.3		1.3±0.1	17.3±0.8	33.5±0.6*		0.88±0.2	NS(11.5–1.7)	
LL+Mel	266.8±79.2	306.0±95.4	15.4±1.3*	36.3±0.3		1.0±0.1	16.8±0.2	35.6±0.2		0.64±0.1	17.9±0.2(17.2–18.4)	

LD: photoperiod 14∶10 during pregnancy and post delivery, lights on at 0700 hours; LL: continuous light during pregnancy and post delivery, LL+ M: continuous light during pregnancy+daily dose of melatonin at 1800 hours until delivery followed by continuous light post delivery. LD = 6; LL = 5, LL+ M = 4. A = amplitude, Φ = acrophase,

* P<0.05 vs LD;

** P<0.05 vs Locomotor activity rhythm; Watson Williams test.

**Figure 2:**
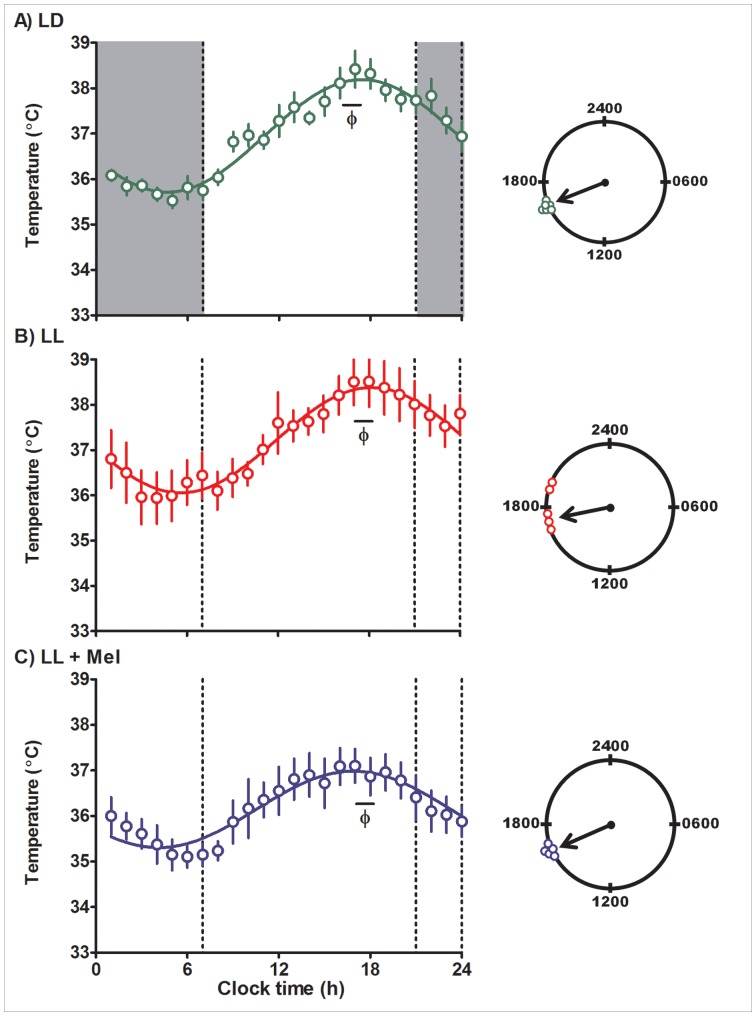
Mean ± SE temperature in pregnant capuchin monkeys 96 hours before delivery. Temperature was measured at 15 min intervals by telemetry. Integrated one h values were used to depict the rhythm. LD: females maintained in light:dark 14∶10 during pregnancy; LL: females maintained in constant light during pregnancy; LL+M: LL females receiving a daily melatonin replacement at 1800 h. Shaded bars represent light off. Φ indicates the acrophase. The continuous line represents the theoretical 24-h cosinor function fitting the data. The clocks at the right of the figure show the distribution of acrophases of the individual temperature rhythms in each group of females. An arrow denotes the timing of the mean acrophase.

Exposure to constant light on the pregnant female had no effect on their circadian body temperature rhythm. In the three groups of pregnant females, acrophases were distributed between 1700–1800 h (P<0.05, Rayleigh’s test for each treatment) and amplitude of the rhythm was similar. Body temperature rhythms were also detected in the mean data by ANOVA for repeated measures (P<0.05). As shown in Figure 2, mean data fitted a cosine function in LD, LL and LL+Mel treated pregnant females (R^2^ 0.96, 0.95, 0.89 respectively). Daily maternal melatonin administration did not change the timing of the acrophases of the activity and temperature rhythms but it slightly decreased the mesor of the maternal temperature rhythm (Table 2). The exposure to constant light altered phase relationship between the locomotor activity and temperature rhythms. Under LD conditions the acrophase of locomotor activity rhythm preceded that of the temperature rhythm. Under constant light the acrophase of both rhythms coincided regardless of melatonin treatment (Table 2).

### Effect of Maternal Chronodisruption during Gestation in the Newborn Temperature Rhythm

**Figure 3:**
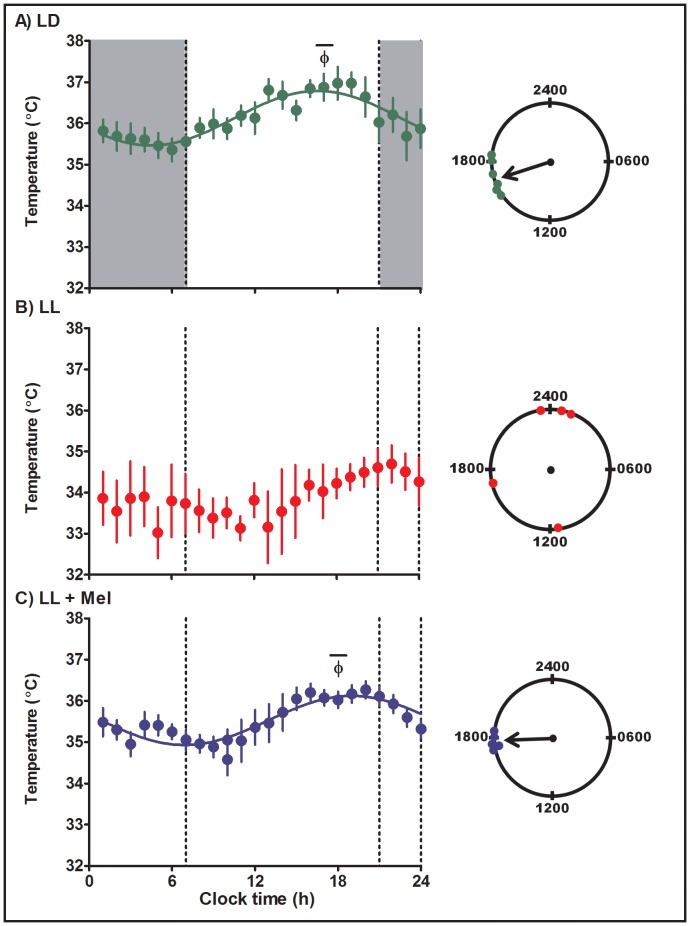
Mean ± SE circadian temperature rhythm in capuchin monkey newborns at 4–6 days of age. Temperature was measured at 15 min intervals by telemetry. Integrated one h values were used to depict the rhythm. LD: newborns from mothers kept in light:dark 14∶10 during pregnancy and reared in LD (n = 6); LL: newborn from mothers maintained in constant light during pregnancy and reared in LL (n = 5); LL+M: newborn from LL mother receiving a daily melatonin replacement at 1800 hours during pregnancy and reared in LL (n = 5). Shaded bars represent light off. Φ indicates the acrophase. The continuous line represents the theoretical 24-h cosinor function fitting the data. The mean data fits a 24-h cosine function (R2 0.83 and 0.82, LD and LL +Mel newborns, respectively).The clocks at the right of the figure show the distribution of acrophases of the individual temperature rhythms in each group of newborns. An arrow denotes the timing of the mean acrophase.

Maternal chronodisruption by exposure to LL brought changes in the newborn temperature rhythm; these changes were reverted by daily maternal melatonin administration (Table 2). Twenty-four hour body temperature rhythms were detected in all individual LD, LL and LL+ Mel newborns by cosinor analysis (Table 2). However, in the LL newborns, the acrophases of the rhythms were randomly distributed over the 24 h. Acrophases in three of the LL newborns clustered around 2400 h whereas in one newborn the acrophase was almost opposite occurring at 1100 h and in the other at 1700 h. In contrast, acrophases of the body temperature rhythm in newborns of mothers kept in LD during pregnancy and reared in LD after birth concentrated in the late afternoon, between 15.8 and 19.1 h, mean 17.0±0.5 h (P<0.05, Rayleigh’s test, Table 2, Figure 3). A second effect of chronic maternal exposure to constant light during gestation was a decrease in mean body temperature (Table 2). Daily maternal melatonin replacement during pregnancy had a synchronizing effect, restoring the acrophase of the newborn temperature rhythm to the late afternoon (range 17.2 and 18.4 h; mean 17.9±0.2, P<0.05, Rayleigh’s test), clock time similar to that found in the newborns from LD mothers and also restored the mesor (Table 2).

The lack of synchronization of temperature rhythms in newborns of mothers exposed to constant light during pregnancy, is also apparent when plotting mean temperature values at hourly intervals over 24-h against clock time (Figure 3). No 24-h changes in the mean data are detected in LL newborns. However a 24-h circadian rhythm of body temperature becomes apparent in the mean data of these newborns when aligning the temperature acrophases of each individual to the same circadian time (Figure S1). In contrast, synchronization of temperature rhythms in LD and LL+ Mel newborns, is evidenced by 24-h changes in the mean data when the data is plotted against clock time (ANOVA for repeated measures and Tukey’s test; Figure 3) and when the data is plotted against circadian time, (Figure S1). Distribution of acrophases of individual newborns in each group is shown in the 24-h clocks of Figure 3.

## Discussion

We investigated in a non-human primate, the capuchin monkey, the impact of maternal chronodisruption during the last third of pregnancy (chronic exposure to continuous light, a treatment that suppresses the maternal melatonin rhythm) on the maternal rhythms of temperature and activity at the end of gestation and its effects on the temperature rhythm of the newborn. Altogether, our data demonstrates persistence of entrained maternal rhythms under constant light, although with a new phase relationship. In contrast, the newborn’s temperature rhythm was greatly affected. Newborns showed a temperature rhythm that was desynchronized and had a lower mesor. Daily administration of melatonin to the pregnant mother, had no effect on the maternal rhythms but synchronized the newborn temperature rhythm and increased the mesor of the rhythm. Thus in a primate newborn, the prenatal information provided by the LD cycle through the daily maternal melatonin rhythm plays a role in newborn temperature regulation, as a prenatal *Zeitgeber* for the phase of the rhythm and by mechanisms not quite understood, in thermoregulatory mechanisms.

There is very limited information on the effects of constant light in pregnant animals, especially in primates. In contrast to what might be anticipated from studies in non-pregnant rhesus and squirrel monkeys under constant light [24]–[25] in pregnant capuchin monkeys maternal chronodisruption by elimination of the LD photoperiod during the last third of gestation did not translate into free-running of the temperature and activity rhythms at the end of gestation. Although these rhythms remain entrained, they displayed a new phase relationship. In constant light, the acrophase of the temperature rhythm was similar to that of pregnant females maintained in LD and of non-pregnant capuchin monkeys [22], whereas the acrophase of the activity rhythm was delayed, coinciding now with that of the temperature rhythm. This contrasts with the 5 hour difference between activity and temperature in LD in pregnant capuchins and in non-pregnant female rhesus monkeys [22], [26]. Also, at difference with reports in non-pregnant rhesus and rats [24], [27]–[28] melatonin treatment did not change the acrophase of the activity rhythm in the pregnant capuchin. It is possible that both entrainment and the shifting of the acrophase of locomotor activity under chronic exposure to constant light may reflect entrainment of the pregnant capuchins to external cues as housekeeping or feeding schedule, that were present in our experimental conditions. The same could apply to the maternal temperature rhythm. The marked contrast between reported responses to constant light in pregnant and non-pregnant monkeys suggests that pregnancy may alter the circadian system response to light. Studies in the rat suggest a functional reorganization of the SCN and ventral subparaventricular zone [29]. An additional study shows differences with non-pregnant rats in c-Fos expression in brain regions controlling sleep and temperature [30]. The persistence of synchronized temperature and activity rhythms in the pregnant capuchin, together with previous data demonstrating that constant light exposure does not affect cortisol, estradiol and progesterone rhythms [20]–[21] may indicate a sort of resilience to perturbation of the circadian system during pregnancy in primates. The relevance of the present findings is to support that most of the putative potential circadian maternal synchronizers for the fetus, such as cortisol, estradiol and progesterone, as well as body temperature and locomotor activity are maintained. However, constant light has a strong direct effect, suppressing melatonin production and thus the melatonin circadian rhythm [20]–[21]. Given our results indicating that melatonin replacement is able to restore the newborn temperature rhythm relative to control LD conditions, this endocrine signal seems to be a major synchronizing cue before and shortly after birth.

Indeed, the absence of a maternal melatonin rhythm during pregnancy had a marked effect on *the newborn temperature rhythm*. A 24-h temperature rhythm was detected at 4–6 days of age in all newborns studied regardless of the prenatal treatments, pointing out to the endogenous nature of the rhythm. However, in LL newborns, the temperature rhythm was not synchronized and temperature mesors were about 2°C lower than in LD newborns. These two effects of exposing the mothers to constant light during pregnancy were reverted in newborns of LL mothers receiving melatonin, again supporting that the gestational maternal melatonin rhythm conveys a photoperiodic signal required to fully achieve synchronized perinatal circadian rhythms. While in utero, the newborns of mothers maintained in constant light during gestation, as well as those LL+Mel mothers were exposed to similar maternal activity/rest and temperature rhythms. After birth both groups of newborns were reared in constant light to avoid providing an LD entraining signal. Altogether, these data demonstrates that in absence of a melatonin rhythm during pregnancy, neither the maternal activity nor the maternal temperature rhythms provide a signal strong enough to entrain the temperature circadian rhythm in the newborn. Evidence from our laboratory suggests that melatonin may have acted at the fetal SCN level. The fetal capuchin SCN expresses the MT1 melatonin receptor and shows oscillatory expression of clock genes synchronized to the LD cycle [31]. Maternal exposure to constant light shifts oscillatory expression of clock genes in the fetal SCN. As in the present report, daily administration of melatonin to the pregnant mother reversed the effect of constant light [31]. Thus we conclude that akin to findings in rodents for behavioural rhythms [15] the daily administration of melatonin to the mother during the last third of pregnancy, acted prenatally as *Zeitgeber* for the newborn primate temperature rhythm.

It is becoming apparent that maternal melatonin plays additional physiological roles along fetal development. It is important to keep in mind that the fetal pineal gland does not produce melatonin and therefore, fetal circulating melatonin is of maternal origin. Newborns from mothers chronically exposed to constant light during pregnancy had a lower mean temperature that was normalized in newborns whose mothers received melatonin. Since the 24-h mean (mesor) temperature rhythm represents the balance of heat production and dissipation, some of these mechanisms were altered by prenatal melatonin deprivation, possibly at the level of brown adipose tissue. Functional melatonin receptors are present in several fetal organs including brown adipose tissue [32]. This tissue is accrued during fetal life in human, non-human primates and sheep and produces about half of the heat needed by newborns to face the transition from the *in utero* environment (over 39°C) to the cooler postnatal environment [33]. Alternatively, prenatal melatonin may act on the pathways involved in heat distribution and conservation [34]. These possibilities are currently being investigated. The former observation added to the involvement of melatonin during gestation in perinatal adrenal function in primates and rats [35]–[37] and the chronobiotic action just demonstrated in the present study, highlights the importance of maternal melatonin during pregnancy for the newborn. At any rate, the very fact that the newborns from mothers under LL displayed an average body temperature which was 2°C lower than controls, may have profound long term consequences for the newborn.

The orderly day/night alternation is an important signal for normal physiological functions [7]. Regarding the potential relevance of the present findings to human health, it must be kept in mind that exposure to light at night (which effectively suppresses melatonin), most probably is a perinatal environmental risk factor imposed by a modern 24/7 society. In this context, our data demonstrate negative effects of maternal melatonin suppression during pregnancy in the newborn primate, which might carry on into adulthood as abnormal physiological traits. This possibility needs to be seriously considered, since there is a body of evidence suggesting that a deleterious maternal environment increases the risk of developing diseases like diabetes, hypertension, obesity and metabolic syndrome that appear in adult life [38]–[39].

## Supporting Information

Figure S1 **Mean ± SE 24-h changes in body temperature in capuchin monkey newborns at 4–6 days of age.** LD: newborns from mothers kept in light:dark 14∶10 during pregnancy and reared in LD; LL: newborn from mothers maintained in constant light during pregnancy and reared in LL; LL+M: newborn from LL mother receiving a daily melatonin replacement at 1800 hours during pregnancy and reared in LL. Body temperature was measured at 15 min intervals by telemetry. Integrated one h values were used to depict the rhythm. The temperature data of each newborn was aligned assigning the maximal temperature value of each individual rhythm to circadian time 19. *P<0.01 vs 7 hrs (Friedmann and Dunn’s post hoc test).
(EPS)

Table S1 **Parameters (mean ± S.E.M.) of the individual 24-h Cosinor equations describing body and rectal temperature in the newborn Cebus apella.**
(DOCX)
