# Supplementary material for: Impact of Chronodisruption during Primate Pregnancy on the Maternal and Newborn Temperature Rhythms
Source: PLoS One. 2013 Feb 28;8(2):e57710. doi: 10.1371/journal.pone.0057710 (PMC3585206; doi:10.1371/journal.pone.0057710)
Supplement: Table S1 — Parameters (mean ± S.E.M.) of the individual 24-h Cosinor equations describing body and rectal temperature in the newborn Cebus apella. (DOCX) [file pone.0057710.s002.docx]

Supplemental Table 1. Parameters (mean ± S.E.M.) of the individual 24-h Cosinor equations describing body and rectal temperature in the newborn *Cebus apella*.

| Measurement Age | Newborn | Mesor (ºC) | Amplitude (ºC) | φ (hours) | P |
| --- | --- | --- | --- | --- | --- |
| Body T 4 – 6 days | 1 | 35·83 ± 0·10 | 0·64 ± 0·02 | 18·6 ± 0·2 | <0·01 |
|  | 2 | 36·06 ± 0·11 | 0·53 ± 0·02 | 18·1 ± 0·2 | <0·01 |
|  | 3 | 35·45 ± 0·10 | 0·99 ± 0·02 | 16·6 ± 0·1 | <0·01 |
|  | 11 | 36·94 ± 0·09 | 0·56 ± 0·01 | 18·3 ± 0·1 | <0·01 |
|  |  |  |  |  |  |
|  | Grand Mean | 36·07 ± 0·32 | 0·68 ± 0·11 | 17·9 ± 0·7 |  |
| Rectal T 4 – 6 days | 1 | 36·62 ± 0·10 | 0·76 ± 0·15 | 18·4 ± 0·7 | <0·01 |
|  | 4 | 36·36 ± 0·05 | 0·40 ± 0·05 | 15·5 ± 0·9 | <0·01 |
|  | 5 | 36·58 ± 0·04 | 0·29 ± 0·03 | 19·6 ± 1·3 | 0·02 |
|  | 6 | 37·56 ± 0·04 | 0·16 ± 0·05 | 18.6 ± 1·2 | 0·01 |
|  | 7 | 36·52 ± 0·07 | 0·54 ± 0·06 | 18·9 ± 0·9 | <0·01 |
|  | 8 | 37·20 ± 0.06 | 0·27 ± 0.04 | 16·8 ± 1·1 | <0·01 |
|  | Grand Mean | 36·81 ± 0·19 | 0·40 ± 0·09 | 18·0 ± 1·4 |  |

φ =acrophase, Body T = temperature measured by radiotelemetry for 24-h. Rectal T= rectal temperature measured at hourly intervals for 24-h. Newborn 1: rectal temperature was measured while recording body temperature by radiotelemetry.
